# Nanomaterials in Dentistry: State of the Art and Future Challenges

**DOI:** 10.3390/nano10091770

**Published:** 2020-09-07

**Authors:** Victoria Bonilla-Represa, Camilo Abalos-Labruzzi, Manuela Herrera-Martinez, M. Olga Guerrero-Pérez

**Affiliations:** 1 Departamento de Operatoria Dental y Endodoncia, Universidad de Sevilla, E-41009 Sevilla, Spain; vbonilla@us.es (V.B.-R.); manuelah@us.es (M.H.-M.); 2 Departamento de Materiales Dentales, Universidad de Sevilla, E-41009 Sevilla, Spain; cabalos@us.es; 3 Departamento de Ingeniería Química, Universidad de Málaga, E-29071 Málaga, Spain

**Keywords:** dental materials, nanomaterials, zeolite, graphene, nanoparticles, nanofibers

## Abstract

Nanomaterials are commonly considered as those materials in which the shape and molecular composition at a nanometer scale can be controlled. Subsequently, they present extraordinary properties that are being useful for the development of new and improved applications in many fields, including medicine. In dentistry, several research efforts are being conducted, especially during the last decade, for the improvement of the properties of materials used in dentistry. The objective of the present article is to offer the audience a complete and comprehensive review of the main applications that have been developed in dentistry, by the use of these materials, during the last two decades. It was shown how these materials are improving the treatments in mainly all the important areas of dentistry, such as endodontics, periodontics, implants, tissue engineering and restorative dentistry. The scope of the present review is, subsequently, to revise the main applications regarding nano-shaped materials in dentistry, including nanorods, nanofibers, nanotubes, nanospheres/nanoparticles, and zeolites and other orders porous materials. The results of the bibliographic analysis show that the most explored nanomaterials in dentistry are graphene and carbon nanotubes, and their derivatives. A detailed analysis and a comparative study of their applications show that, although they are quite similar, graphene-based materials seem to be more promising for most of the applications of interest in dentistry. The bibliographic study also demonstrated the potential of zeolite-based materials, although the low number of studies on their applications shows that they have not been totally explored, as well as other porous nanomaterials that have found important applications in medicine, such as metal organic frameworks, have not been explored. Subsequently, it is expected that the research effort will concentrate on graphene and zeolite-based materials in the coming years. Thus, the present review paper presents a detailed bibliographic study, with more than 200 references, in order to briefly describe the main achievements that have been described in dentistry using nanomaterials, compare and analyze them in a critical way, with the aim of predicting the future challenges.

## 1. Introduction

**Figure 1:**
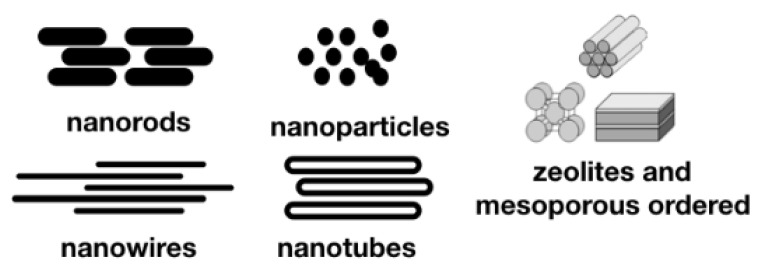
Scheme of the shape of the nanomaterials most useful for medicine.

Zeolites and other ordered porous materials, including nano-shaped carbon materials, are those in which the surface composition, as well as the porosity and/or shape, are controlled at a nanometric scale. By this manner, it is possible to control several physical and chemical properties, such as the shape/structure at a molecular level, the high surface area and adsorption capacity, ion-exchange ability, uniform porosity, and accessible pore volume. By controlling these properties, it is possible to design functional materials with important applications in energy, environment and medicine. The most important families of porous nanomaterials are zeolites, metal organic frameworks (MOFs) and nanostructured carbons. Due to the important properties that these materials have, during the last decade, several steps in the design and application of porous nanomaterials have been taken and several commercial applications are available in the fields of batteries, CO_2_ capture, catalysis, environmental applications, adsorbents, etc. [1,2,3]. Nanotubes, nanorods and nanowires (Figure 1) are also nanostructured materials in which the shape and the surface composition are controlled.

These materials present advantages for their use in different biomedical applications since they allow performing molecular-scale medical interventions for treating several diseases or for repairing damaged tissues, and the sizes of the porous/nanoparticles being in the range of the biomolecules. The possibility of controlling the pore volume, shape and diameter, along with the possibility of modulating the surface properties to increase solubility, immunocompatibility and cellular uptake, open the possibility of synthesizing hundreds of nanodrugs with several applications [4]. Through this method, nanomedicine is being developed [5], opening new applications and possibilities. This new concept of medicine is offering new therapies for cancer [6,7], including nanodrugs for killing cancer cells and new methods of cancer tumor detection. The porous nanomaterials can be used as containers for the agent responsible for killing the cancer cell, which can be inside the pore and be released when it reached the cancer cell, or it can be attached to the surface of the material by functionalizing it. The possibility of the functionalization of the surface of the material can confer to it certain magnetic [8,9] or acid-based properties that can be used for forcing the release under controlling conditions. In addition, functionalization is used for including biochemical markers able to detect tumoral cells. These drug delivery properties are also used for the local administration of drugs for several infectious diseases. The local administration of drugs presents several advantages, such as the use of a lower amount of drug, decreasing the side effects in the healthy cells. The applications of these materials are wide, since they can be used to deliver small-molecule drugs as well as various classes of biomacromolecules, such as peptides, plasmid DNA, proteins, and oligodeoxynucleotides. The properties of nanomaterials make them also suitable for the design of new tissue engineered materials, whereas traditional tissue engineering was based on hydrolytically degradable macroporous materials, and current synthesis approaches emphasize the control over the cell behavior and tissue formation at the nano-scale [10].

**Figure 2:**
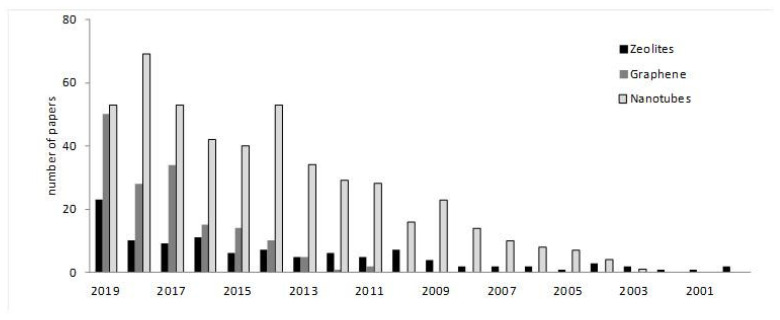
Tentative number of papers published during the last two decades. The number of papers has been calculated on the basis of a search including each word (zeolite, graphene or nanotubes) and dental or dentistry, according to Scopus.

Despite the revolution that is happening in the medical field due to the development of nanomaterials [11,12], their applications in dentistry mostly remain to be explored, this being a field that will undoubtedly develop a lot in the coming years, modifying the actual protocols for drug delivery and tissue engineering systems in dentistry. To give an idea about this issue, the number of papers during the last 20 years that appeared in Scopus for “zeolite”, “graphene” or “nanotubes” with “dental” or “dentistry”, are plotted in Figure 2. It is interesting to note the low amount of papers in which zeolite applications have been explored, and if the search is done with other families of porous materials, such as MOFs, is done, the number of papers is negligible, indicating that the applications in dentistry of these nanomaterials are not very developed. The search data (Figure 2) indicate that more research efforts have been done with nanotubes and graphene applications, although it can be observed how the interest in graphene has been growing during the last 3–4 years, whereas the number of papers for nanotubes during the last 4–6 years seem to be stable. Thus, since the application of these materials in dentistry are just starting to be explored, it is the aim of the present contribution to review them to date, in order to clarify the work that has been reported in these areas, and which are the future trends. Metal nanoparticles could also be considered nanomaterials, but their properties and applications are different from the nano-shaped nanostructures, which are the main focus of the present paper.

## 2. Zeolites

Due to the fact that it is possible to control the pore or pore diameter of zeolite-based materials, and also the active sites and adsorption properties, they have been extensively used as adsorbents and as catalysts at the industrial scale [13,14], and subsequently their properties have been properly described. In recent decades, synthesis methods of zeolite nanomaterials have been described [15], including stable colloidal suspensions [16], zeolite nanocrystals [17], zeolite nano and microfibers [18], and zeolite thin films and membranes [19]. Due to all this research effort, it has been possible to prepare materials in which the pore diameter and surface adsorption properties, as well as the material nano-shape, can be modulated. These materials found several applications in biotechnology and medicine, such as controlled drug and gene release, the separation of biomolecules and cells, the improvement of the nutrition status and immunity of farm animals, biosensor applications, and the detection of biomarkers of various diseases, radical scavenging, and tissue engineering and biomaterial coating [20]. In dentistry, zeolite materials have mainly been investigated for their uses in obturation, endodontics, and prostheses, although the number of papers on zeolites in dentistry is quite low (Figure 1), indicative that this is still a field to be developed.

Okulus and coworkers [21] described the use of LTA-type (Linde type A) zeolite fillers for resin-based dental materials with remineralizing potential. LTA-type zeolite was used due to its ability to incorporate Ca^2+^ cations, which are considered to have anticaries activity, since there are able to rebuilt the HA (hydroxyapatite), Ca_10_(PO_4_)_6_(OH)_2_, structure [22]. Some studies had already described the use of zeolite–HA composites as promising bone tissue engineering applications [23] due to their biocompatibility, stability, and activity in the proliferation of the normal human osteoblasts. In the cited study [21], several parameters were evaluated for the synthesis of LTA zeolites. Then, these materials were subjected to an ion exchange process with CaCl_2_. These materials were incorporated to an organic matrix to form ionomers. Although the Ca^2+^ release capability is lower for the composites when they are compared with the Ca–zeolite materials, it was comparable to the cation release that showed other materials, such as calcium phosphate-filled and glass ionomer cements. Thus, this study [21] confirmed that this is a promising application in dentistry for zeolite-based materials, although more research efforts are necessary to improve the Ca^2+^ release properties of the zeolite-containing composites. Kim and coworkers [24] described a similar approach, although not with Ca^2+^. They used the zeolite material as carrier for chlorhexidine, which was incorporated into commercial dental glass ionomer cement. This way, the antimicrobial properties of the cement were improved.

Although those few examples described in the previous paragraph contemplated the use of zeolites as carriers for Ca^2+^ cations and chlorhexidine, in most cases, the use of zeolites in materials for fillings and endodontics is a support for Ag nanoparticles. The antimicrobial effects of metallic silver and its salts are well known [25], and many recent studies [26] have focused on the use of Ag nanoparticles. Depending on the application, some authors relate the microbial effect to the shape of those nanoparticles [27,28], and some to the microbial activity of Ag^+^ cations [29]. The use of silver nanoparticles is quite promising in several medical applications, including dentistry [30], since several pathogenic bacteria have developed resistance against various antibiotics, and in addition, nanomaterials are allowing the development of silver-based dressings, coatings, and silver-coated medicinal devices such as nanogels and nanolotions [31,32]. Sinanen Zeomic, commercialized in 1984 as Zeomic^®^, is a zeolite material doped with silver, and according to the commercial specifications, able to release Ag^+^ cations. This antimicrobial commercial agent has been used in some research studies in dentistry. For example, Nakanoda and coworkers [33] evaluated its antifungal effect against acid production (and/or Candida albicans growth), by combining commercial Zeomic^®^ with a tissue conditioner. Their results with a 4–5% Zeomic^®^ loading showed a significantly greater effect on the pH value decrease when compared with the unloaded samples, suggesting that these materials can have some applications in denture stomatitis. Several studies [34,35,36] also show that zeolite loaded with Ag ^+^ cations is a good additive to Mineral Trioxide Aggregate (MTA) since it is able to confer it with an antimicrobial effect, finding a correlation between the amount of Ag+ cations released and the bacteria/fungi growth inhibition. MTA is a cement widely used in endodontics [37], since it is biocompatible, insoluble in tissue fluids, and able to seal the pathways between the root canal system and its surrounding tissues. All these examples show that Ag-loaded-zeolite materials are very useful as an additive for filling materials and cements and in addition to their antimicrobial activity, it has been reported that the incorporation of the zeolite does not affect certain tissue conditioner’s dynamic viscoelastic properties [38] and that the antimicrobial effect is not influenced by saliva for at least one month [39]. It has also been reported that MTA doped with Ag/zeolite, presents a higher antimicrobial activity than an MTA treated with chlorhexidine [40]. In addition to Ag, it has been reported that Cu and Zn salts and nanoparticles [41] are also active to fight the growth of bacteria, alone or in combination with silver. In this sense, Samiei and coworkers [42] used a conventional ZSM5 type zeolite, loaded (following an ion exchange procedure) with Ag^+^ and Zn^2+^ cations. Although the antimicrobial effect that they conferred to the MTA cement was desirable, they reported a decrease in the compressive strength of the material, limiting its applications.

A common problem regarding dental resins is that they have a water sorption capacity that can reach 2.5–3% [43]. This sorption capability, along with their heterogeneous surface after polymerization, makes them vulnerable to surface fouling by microbiotes such as Candida, which can form a biofilm on the resin surface [44]. To solve this, Tosheva et al. [45] proposed the introduction of Ag/zeolite in dental acrylic resins. They prepared a conventional faujasite zeolite, followed by an ion exchange procedure for Ag^+^ introduction. The impregnation of this Ag/zeolite material into the resin was made by incubation at room temperature for 24 h under continuous stirring. In their study, it was demonstrated that the antimicrobial activity was correlated with the Ag^+^ release capability, and that such activity was lost after 60 days of incubation in aqueous media. These results are in line with those reported by Saravanan et al. [46], which evaluated in vivo the antimicrobial effects and viscoelastic properties of Ag/zeolite-doped dental acrylic resins. However, it was reported by Tosheva et al. [45] that the introduction of Ag/zeolite to the resin did not affect the appearance of the material and the mechanical properties were within the standard requirements, although other authors have reported a decrease in the flexural and impact strength values of the acrylic resins after Ag/zeolite doping [47].

There are also some studies that have explored the incorporation of zeolite into titanium alloys, which are widely used in dental and orthopedic implants. Despite their biocompatibility and corrosion resistance, Ti alloys can release some V and Al ions, as has been reported from some in vitro studies [48], causing poor osseointegration and limiting the lifespan of the Ti prosthesis. To avoid such Al and V ion release, a zeolite coating deposited on the Ti alloy has been proposed [49]. In this manner, the dissolution of the alloy metals is prevented and the modulus mismatch with bone issue is reduced, enhancing the osseointegration. Thus, this study showed that zeolite coatings are promising for hard tissue regeneration applications. In line with this study, more recent papers [50] also demonstrated that the use of zeolites can improve the osseointegration of Ti alloys. Other authors have proposed a similar procedure but with a Zn-containing coating, by the use of a ZIF (zeolitic imidazolate framework) material. This way, and in addition to the osseointegration capability described for the zeolite coatings [48,49], the release of Zn^2+^ cations confer to the Ti alloy antimicrobial capability [51].

It has been shown that zeolites found many important applications in dentistry, as is summarized in Table 1. Due to its capacity to retain cations and molecules by ion exchange/impregnation and subsequent release, they have been described several applications depending on the molecule/cation: Ca^2+^ (remineralizer), Ag^+^ (antimicrobial), and chlorhexidine (antimicrobial). In addition, zeolite coatings can improve the osseointegration capability of Ti alloys. These studies show that zeolite materials are quite promising in dentistry, but the number of studies about applications of zeolite-based materials in dentistry, in comparison with other fields in biotechnology and medicine, is low. Thus, this is indicative that this field of research must be developed in the incoming years.

## 3. Graphene

Graphene consists in a single layer of carbon atoms with a hexagonal honeycomb lattice that was isolated for the first time in 2004 by Geim and Novoselov at The University of Manchester. Such structure makes graphene the thinnest known material, and also confers it some extraordinary properties such as a very high mechanical strength, electricity and heat conduction, having no effective mass [52]. Since its discovery in 2004, this innovative and revolutionary material has opened many lines of research and is revolutionizing areas such as precise sensors, solar panels, faster electronics, coatings, paints, and of course medicine. In addition to graphene, 2D analogues have been described, also with an ultrathin-sheet structure, but with another chemical composition such as graphitic carbon nitride, transition metal oxides and dichalcogenides, or boron nitride [53]. Graphene-based materials and their analogues have been demonstrated to find important applications in nanomedicine and nanobiotechnology, such as gene transportation, anticancer drug release, photothermal and photodynamic therapies, biosensors and tissue engineering [54], most of them being evidently quite relevant in dentistry. When graphene is rolled up, a carbon nanotube is obtained, thus carbon nanotubes and graphene-based materials have some similarities, but in order to analyze the applications in a systematic way and for the sake of simplicity, applications with graphene materials will be discussed in the present section whereas carbon nanotubes will be analyzed in detail in the next section, with some other kind of nanotubes and nanofibers.

One of the most explored applications of graphene-based materials in dentistry is for tissue engineering [55] and pulp-denting regeneration [56], as a smart reinforcing scaffold material [57,58,59,60]. The most common available procedures for bone regeneration (allograft, isograft, autograft and xenograft) have many potential risks, notably in developing adequate bone regeneration therapies. This is due to the properties of graphene family materials that make them suitable for the structural reinforcement of hydrogels, films and other scaffold materials that are commonly used for tissue engineering [61]. Graphene-based materials have been demonstrated to increase the strength, elasticity and mechanical properties when they are added to the most common materials used for tissue engineering, such as hydrogel composites made of synthetic hydrophilic polymers including polyvinyl alcohol and poly (methyl methacrylate), or chitosan gels. In addition, it has also been proved the improvement of the osteogenic potential of graphene-coated surfaces. This is a very interesting application of graphene-based materials, since hydrogels have very weak mechanical properties that limit their use in many tissue engineering applications [62]. In addition to its mechanical and electrical properties, graphene functionalization with protein/peptides will be useful for tissue engineering applications [63]. For example, Kawamoto and coworkers [64] prepared graphene oxide scaffolds (GO). They demonstrated that they presented a quite low cytotoxicity and that they were able to enhance the cellular ingrowth behavior. They also demonstrated that this material was able to increase the periodontal attachment formation, cementum-like and ligament-like tissue, in comparison with the scaffold that was not doped with graphene oxide. These applications of graphene and its derivatives in dentistry are quite relevant, especially for implants, membranes, and cements, in addition to other applications that have been explored, such as teeth whitening, bacteria treatment, and biosensors. Another example can be found in the study reported by H. S. Jung and coworkers [65], who prepared a Ti alloy with osteogenic dexamethasone that was loaded in a graphene material. Such material resulted in a significant increase in the differentiation of the growth of osteoblasts.

Titanium is the most common material to be used in implantology, mainly due to its biocompatibility. However, this material presents some disadvantages, since it has been detected that it can generate alloy particles and ions into surrounding tissues, which result in bone loss and the osseointegration failure of the implant [66]. Therefore, Ti materials are the best option nowadays, but they still need to be improved. Graphene oxide (GO) is the material that has been more explored in this field. It is composed by the exfoliated monolayers of a few-layered stacks of graphite oxide. These layers are approximately 1 nm thick and 400–500 mm long, having a very high aspect ratio [67]. Several groups have investigated the GO coating of Ti implants in order to improve the osseointegration since it is biocompatible, has antibacterial properties [68] and can enhance the mechanical properties [69]. In addition, GO coatings prevent corrosion [70]. Nishida et al. [71] reported the fabrication of a GO-applied scaffold and showed through in vivo studies that such scaffolds were able to enhance new bone formation. Mohammadrezaei and coworkers performed a systematic literature review to uncover the parameters’ effect on bone regeneration [72] in order to establish some parameters that improve osseointegration in a safer way. They concluded that a mass ratio ≤1.5 wt% for all and a GO concentration up to 50 μg/mL can be considered safe for most cell types, although the maximum concentration depends on the cell type. In this line, Gu et al. [73] grew single layer graphene sheets on Ti by chemical vapor deposition (CVD) and evaluated the effect of thermal treatment (2 h at 160 °C) after the CVD in order to increase the adhesion strength and osteoinductive activity. They demonstrated that thermal treatment enhanced adhesion and did not affect the favorable effects such as osteogenic differentiation and antibacterial activity. Suo and coworkers prepared a GO/chitosan/hydroxyapatite (HA) composite coating that was deposited on the Ti material by electrophoretic deposition [74]. The objective of the cited paper was to improve the properties of HA coatings since, although they have been demonstrated to improve the osseointegration between the implant and the bone [75], it possesses a quite low mechanical strength that limits its use. The addition of chitosan can improve the coating adhesion on the Ti surface of the HA coating and facilitates the osseointegration. It was demonstrated [74] that cell viability, cell differentiation and cell mineralization were significantly enhanced with respect to the reference coatings (those that did not include HA, chitosan and GO). In addition, the biomechanical properties of the new bone formed in vivo around the GO/chitosan/HA implant were also enhanced, as was demonstrated through the animal study. Another approach that used tea polyphenol-reduced oxide (TPG) instead of GO was reported by Liu and coworkers [76]. They deposited a TPG layer on the Ti material by electrochemical deposition. The in vivo results reported proved that the TPG layer was able to improve the osseointegration.

Another important issue when adding graphene to Ti implants is the antibacterial activity that graphene confers [77,78,79,80,81]. Several studies can be found elsewhere showing that GO incorporation into resins and membranes prevents the microbial adhesion [82,83,84] of several common mouth microbes [85]. This effect is related to the physical damages in bacterial membranes that graphene can cause with its sharp edges and the destructive extraction of lipid molecules [86,87]. It can also be improved by the functionalization of the graphene layer with other antimicrobial agents, such as silver nanoparticles [88,89,90]. The functionalization can be used, in addition to the antibacterial applications, for the delivery of many drugs. For example, La and coworkers [91] demonstrated that GO can be an efficient carrier to deliver proteins, another application in implantology. They coated Ti material by a layer-by-layer assembly of positively and negatively charged GO sheets (GO–NH_3_^+^ and GO–COO– respectively). They loaded a therapeutic protein (bone morphogenetic protein 2) on the Go–Ti material and demonstrated, through in vitro human bone marrow-derived mesenchymal stem cells tests, that the osteogenic differentiation was higher when the cells are cultured on Ti–GO instead of Ti, showing how GO on Ti is an effective carrier for the controlled delivery of therapeutic proteins.

Graphene coatings are also able to enhance the proliferation of dental pulp steam cells [92,93]. Rosa et al. [94] evaluated the cytocompatibility and differentiation potential of dental pulp stem cells on GO substrates and concluded that cells are able to attach satisfactorily to the substrate, as well as their proliferation. They also showed that GO increased the expression of some genes involved in the upregulation of mineral-producing cells. Rodriguez-Lozano [95] and coworkers proposed composite films of GO and silk fibroin to improve the cell proliferation and viability. One application was described by Di Carlo and coworkers [96], whom prepared GO-coated membranes (Lamina^®^), and the in vitro analysis demonstrated that the GO coating favored the proliferation of stem cells and promoted the adhesion. Xie et al. [97] demonstrated that graphene induced a high level of mineralization as compared to glass, as was evaluated through pulp stem cells cultures. They concluded that graphene was able to induce osteogenic and not the odontoblastic differentiation of dental pulp stem cells.

The addition of graphene to membranes in oral surgery is useful to prevent soft tissue cells from infiltrating the growing bone [98]. Several authors have investigated the effect of GO addition to collagen membranes, and culture experiments showed that GO addition prevented any type of inflammatory response, and overall, favored the proliferation of human gingival fibroblasts [77]. In addition to membranes, it has also been investigated as an additive for resins and cements. Traditional polymeric and composite materials have some disadvantages that could be overcome by the use of graphene, such as bacterial adhesion and the formation of biofilms [99]. Duvey and coworkers [100] studied the incorporation of graphene sheets to calcium silicate cements, in order to improve some of the disadvantages that these materials have, such as mechanical properties and long setting time. In the same line, Bacali et al. [101] studied the incorporation of graphene into polymethyl methacrylate resins. In this case, they incorporated graphene sheets doped with Ag nanoparticles in order to increase the antibacterial properties. They described how Ag–graphene sheets improved the water absorption and mechanical properties of the resins. Bregnocchi and coworkers [102] reported the use of graphene as fillers for polymer dental adhesives and reported the superior antibacterial activity of such materials with similar mechanical properties (for a 0.2 wt% graphene concentration). In this case, they described 4% graphene as the ideal concentration to reach such objectives. Graphene is also beneficial as the doping of alloys, as demonstrated by Rokaya et al. [103], whom added Ag nanoparticles/GO layer to a NiTi alloy by electrophoretic deposition, in order to improve the mechanical properties and antibacterial of the alloy. A similar study with glass ionomer cements was performed by Sun et al. [104], which used fluorinated graphene with the objective of improving the antibacterial properties of the cement, and in addition, conferred a fluoride ion-releasing property to this material. Nam and coworkers [105] also used fluorinated graphene as a doping agent for orthodontic bonding resins, in order to prevent white spot lesions, due to the antibacterial activity and remineralization effect that is conferred to the resin. Polymethyl methacrylate (PMMA) bone cements are also very frequent in dentistry and medicine, and graphene is also a useful candidate to increase their resistance to mechanical fatigue and impact. Paz et al. [106] studied whether the addition of graphene affected other important properties, such as the thermal ones (thermal conductivity, heat generation, as the extent of the polymerization reaction and glass transition). They satisfactorily concluded that the addition of GO to PMMA cements did not significantly affected thermal properties.

Graphene in biomedicine can be used as an optical biosensing platform [107]. In this sense, Li and coworkers [108] described the interactions of graphene quantum dots with the dental pulp stem cells in order to use them as a fluorescent labeling of stem cells. This would offer valuable information after the transplantation for evaluating the efficacy of stem cell therapies. Son and coworkers [109] reported recently that a GO quantum dot coating can be an effective treatment for dentin hypersensibility, due to its mineralization activity and capacity for dentinal tubule sealing. On the other hand, it is well known that hydrogen peroxide (H_2_O_2_) is commonly used for teeth whitening treatments since it is able to penetrate the layers of the enamel, oxidizing some of the compounds that cause discoloration. The oxidation reaction mechanism is catalyzed by the hydroxyl radicals (OH) that are produced when the peroxide is decomposed. Such decomposition can be enhanced by ozone or UV-light, which is commonly used in the professional teeth whitening treatments. The peroxide decomposition to form radicals can also be catalyzed with metal salts, something frequently used in the oxidation-based processes for removing pollutants, such as the Fenton oxidation processes, in which Fe is used [110], although other metals can also catalyze these oxidation processes [111,112]. Following this idea, Su and coworkers [113] described the use of a cobalt–tetraphenylporphyrin/reduced graphene oxide as a nanocomposite for catalyzing the peroxide decomposition during teeth-whitening treatments.

**Figure 3:**
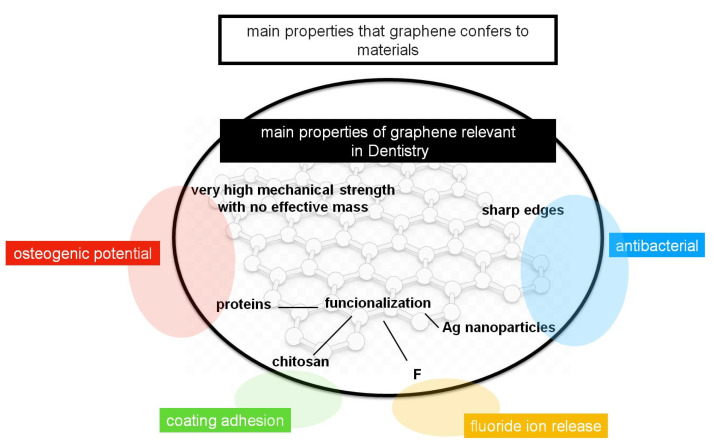
Main properties of the graphene-based materials that are relevant in dentistry (inside circle), and the main properties that they confer to the materials that are commonly used in dentistry (outside circle).

It has been shown that graphene-based materials, and mostly graphene oxide (GO), have many properties that made them quite relevant as dopants for materials that are used commonly in dentistry, as is summarized in Figure 3. Graphene-based materials confer a very high mechanical strength with no effective mass, in addition to antibacterial activity, due to its sharp edges, that can be enhanced with a functionalization with Ag nanoparticles or other drugs. In addition, the coating adhesion can be increased with the functionalization with chitosan, as well as the functionalization with proteins enhances the properties of the graphene regarding the bone regeneration and osteogenic potential. Thus, all these potential properties made graphene and its derivatives useful dopants and coating agents for Ti implants, alloys, adhesives, composites, membranes, resins and cements.

## 4. Nanorods, Nanowires, and Nanotubes

Nanorods, nanowires and nanotubes were cylindrical-shaped nanomaterials. Nanowires usually present a very high length to width ratio with respect to nanotubes and nanorods. Nanorods and nanowires are usually synthesized from semiconducting metals or oxides, whereas the term nanotube usually refers to those shapes that are empty (a tube), that can be made from metals and frequently from carbon. Nanotubes can be made with a single wall or with multiple walls. When they are made from carbon they are named as single-wall carbon nanotubes (SWCNT) or as multi-wall carbon nanotubes (MWCNT) (Figure 1). In addition, nanofibers, that are fibers with diameters in the nanometer range, and that can be considered between nanorods and nanowires, can be included in this family of nanomaterials. These materials have found many applications in many fields, such as energy conversion and storage [114,115,116], catalysis [117] and photocatalysis [118], biotechnology and medicine, for applications such as drug delivery, tissue engineering [119] and cancer diagnosis [120].

As it has been discussed for graphene and its derivatives, carbon nanotubes have a very high mechanical strength that made them quite advantageous for many applications in the synthesis of materials for dental applications. As with graphene derivatives, one of the main applications of carbon nanotubes is tissue engineering, as scaffolds to provide a suitable environment for the incorporation of cells, or for growing factors in order to regenerate damaged tissues [121]. In this sense, de Vasconcellos et al. [122] reported the synthesis of a biomaterial from MWCNT and hydroxyapatite, and its application as a bone substitute to improve regeneration in interventions requiring mesenchymal stem cell differentiation into osteoblast for healing. Terada et al. [123] prepared a MWCNT coating on titanium, reporting a good cell proliferation and strong adhesion. Following this line, Nahorny and coworkers [124] prepared a MWCNT/GO material combined with hydroxyapatite, which resulted as useful as a protective coating for present dentin erosion. With a similar approach, Meng et al. [125] prepared a nydorxyapatite/MWCNT composite, reporting a very high mechanical strength and fracture toughness. In another application, a SWCNT-based material proved to be useful for the differentiation of stem cells from dental tissues (apical papilla [126]. Wang et al. [127] reported a MWCNT-doped polycarbosilane composite, prepared by spark plasma method, that had SiC nanoparticles. Such material presented good mechanical properties for bone tissue and dental implants, and the authors proposed such a composite as a candidate dental implant material in the future. A similar approach was adopted by Chew and coworkers [128] which reported the synthesis of a calcium phosphate cement doped with MWCNTs for bone substitute applications. Carbon nanotubes can also be used to prepare smart coatings for the surface of Ti implants. In this line, Mekhalif et al. [129] prepared a Ta_2_O_5_/MWCNT composite coating on the surface of Ti implants by a sol–gel process. Ta_2_O_5_ was selected because it is biocompatible and quite resistant to corrosion. They concluded that such a coating layer was able to improve the hydroxyapatite formation.

Carbon nanotubes have also been proven to improve the mechanical strength of several materials commonly used in dentistry, similarly to the aforementioned addition of GO. The effect is similar to the usage of steel to increase the hardness of the common cement, forming concrete, due to the bonds formed between cement and steel and the effect that steel scaffolds have in the final structure. Indeed, carbon nanotubes are actually also used for the reinforcement of cementitious composites and concrete in civil engineering [130] and in general, in polymer composites with applications in several fields. The main issue for this application is to achieve the homogeneous dispersion into the polymer matrix, which can be overcome with solution mixing or melt blending [131]. In dentistry, Marrs et al. [132] studied the addition of MWCNT to a PMMA cement, and concluded that, as in the case of adding GO [106], and as expected, MWCNT materials were able to increase their mechanical properties without affecting the thermal properties. In another work, Bottino et al. encapsulated doxycycline in a nanotube-modified dentin adhesive [133], in order to propose a procedure for the synthesis of therapeutic adhesives. Similar studies were performed with other materials commonly used in dentistry, such as resins, composites and alloys [134,135,136,137]. Thus, both graphene and carbon nanotubes are able to increase the mechanical strength of alloys, resins, polymers and cements in dentistry.

Thus, both carbon nanotubes and graphene present quite similar properties and applications, for instance, graphene is just an unrolled nanotube. The main difference between both structures is the electronic structure. Graphene is a zero-band-gap semiconductor due to its honeycomb structure whereas nanotubes can show either semiconducting or metallic properties depending on the chirality. Both of them act as reinforced scaffolds and they can also be functionalized, both properties very relevant in dentistry with several applications having been explored, as mentioned, although that, some differences are evident between both of them. Due to the shape of both structures, it has a higher number of reactive edge surface sites in the graphene and due to that, several applications in dentistry, and specially in implantology, use the antibacterial properties of graphene without loading with any antibiotic or metal nanoparticles, as shown [77,78,79,80]. The higher bioactivity of graphene with respect to nanotubes was also demonstrated through in vitro photothermal anticancer studies [138]. However, the carbon nanotubes edge sites have some reactivity [139] and some studies have also found some microbial activity in nanotubes [140]. These differences in the reactivity, number and position of active sites are relevant to the functionalization, and the use of any of them shall be considered depending on the application. In a preliminary approach, since graphene usually has more edges than nanotubes, it is easily functionalized. With respect to their use as fillers in polymer and composites, it has been shown that after a limit of loading, there is a decrease in the electrical and mechanical properties due to the agglomeration of the filler (graphene or carbon nanotubes). In this case, the shape of graphene improves these properties, since it can form an intricate conducting network within the polymer host matrix [141].

In addition to carbons, nanotubes can also be prepared from materials such as Ti or a Ti alloy, which are widely used in orthopedic and dental applications, and dental implants with surface TiO_2_ nanotubes coated coverage are commercially available. These Ti-based nanotubes increase the surface roughness of the implant [142,143], improving the osseointegration [144], antibacterial properties [145] and in addition, can be functionalized in several ways [146], opening a wide range of possibilities [147]. For example, Balasundaram et al. [148] prepared TiO_2_ nanotubes by electrochemical anodization, and successfully immobilized a bone morphogenetic protein on them and examined the human osteoblast responses through an in vitro study. They reported the enhancement of osteoblast adhesion in comparison to the non-functionalized nanotubes. In this sense, Kodama and coworkers reported that TiO_2_ nanotubes can also be loaded with hydroxyapatite [149], in order to increase the osseointegration. Cao et al. [150] immobilized peptide sequences on the TiO_2_ nanotubes in order to enhance the osteogenic gene expression, as demonstrated by in vitro tests. TiO_2_ nanotubes can also be functionalized with anti-inflammatory [151] or antibacterial agents [152], metal nanoparticles such as ZnO [153], Au [154], Ag [155], or prepared as an alloy with other metals such as Cu [156] in order to confer them antimicrobial activity. In addition to implantology, where those studies have proven the advantages of using Ti-based nanotubes, there are not many described applications of these materials for other applications, and just a few papers describe the use of TiO_2_ nanotubes as the reinforcement of dental materials. Khaled et al. [157] used TiO_2_ nanotubes by incorporating them in a cement matrix and reported an increase in the mechanical stress. On the other hand, dos Santos et al. [158] evaluated through an in vitro study the incorporation of TiO_2_ nanotubes into zirconia composite surfaces in order to evaluate the effect in the bond strength, reporting that they did not have any beneficial effect.

Similar to other nanomaterials, nano and microfiber are garnering attention since they have potential applications in many fields, such as electronics, catalysis, and of course, dentistry and medicine. Several synthesis methods have been described; among all, electrospinning is the preferred one, since it is non-expensive, simple, and relatively easy to scale [159,160]. In this technique, an electric field is applied to the end of a needle that contains the polymer solution held just by the surface tension. When the intensity of the field is increased, the fluid at the tip of the needle starts to elongate, and when it reaches a critical value, the surface tension forces and a jet of the solution is ejected from the tip, allowing the jet during the process to become very long and thin, forming fibers or wires. Nanorods, that are shorter than fibers, can also be prepared by electrospinning, although other methods such as hydrothermal can also be used. As well as nanotubes, these fibers also can act as scaffolds for tissue engineering, controlled drug release and implants [119,161]. The most investigated application of nanofibers/nanorods in dentistry is with chitosan [162] and hydroxiapatite fibers, whose synthesis by the electrospinning method has also been described [163,164,165], since both materials are well known for their applications in epithelial and bone tissue regeneration, as mentioned. The advantage of the nanomaterial formulation is that the osteogenic and cementogenic differentiation is enhanced due to the nano surface structure [166,167,168,169,170] as well as the stem cell differentiation regulation [171]. Atai et al. [172] prepared hydroxyapatite nanorods by the hydrothermal method and incorporated them into a dentin adhesive. They described that the nanorods were stable in the solution and that they were well dispersed, avoiding particle aggregation, and reported a higher bioactivity in the nanorod-doped adhesive. In addition to these, other synthesis methods, as by sol–gel [173], spray drying [174], microware-assisted [175], sonochemical homogeneous precipitation [176], by a hard-template [177], polyvinylpyrrolidone (PVP)-assisted hydrothermal method [178] have been described.

An interesting application of hydroxyapatite nanorods was described by Clarkson et al. [179], who modified the surface of the nanorods depositing a monolayer of surfactant in order to allow then to assemble into an prisme-like enamel structure at the water/air interface, with the objective of mimicking the natural biomineralization process to create dental tissue enamel. In addition, in this line of enamel applications, the same group [180] described the synthesis of fluorapatite nanorods and nanowires, in order to incorporate them into dental materials for caries prevention treatments. More recent works have also focused on the synthesis of hydroxyapatite nanomaterials for the remineralization of enamel [181,182,183,184,185,186,187] as well as in the preparation of fluoridated hydroxyapatite nanorods [188].

Hydroxyapatite nanorods/fibers have been also described as efficient reinforcement materials for composite resins and polymers [189,190], with the advantage of the high remineralization capability. Hydroxyapatite nanorods can present also antimicrobial properties, for example, Chen and coworkers [191] loaded the nanorods with zinc particles, so the material was able to load Zn^2+^ upon use, presenting a high activity inhibiting oral cavity bacteria. In the field of tissue engineering, some applications have been explored, for example, Ren et al. [192] described polyvinyl alcohol collagen hydroxyapatite, Zhu et al. [193] a CaP-hydroxyapatite, and Asran et al. [194] PVA–hydroxyapatite, in all cases nanofibers composites, in both cases by electrospinning and also in both cases it demonstrated the potential of such fibrous materials as bone tissue scaffolds. A similar approach with chitosan has also been described recently [195].

All these works show the potential of hydroxyapatite nanostructures due to their attractive bioactivity and biocompatibility, however, they are limited due to their low mechanical strength, although there are some ways to overcome this issue. In this sense, Mangalaraj and coworkers [196] described that hydroxyapatite nanorods can be reinforced with polyethylene, that could be useful when they are used as reinforcement materials as well as tissue scaffolds. TiO_2_–hydroxyapatite nanocomposites are also quite relevant in dentistry and it is a good option to combine the properties of both materials [197], in this sense, several authors have described the synthesis of TiO_2_ materials doped with hydroxyapatite nanorods [198,199,200,201], demonstrating that the mineralization capacity is enhanced.

Carbon nanotubes and polymer nanofibers, due to their shape and their ultrasmall diameter, are ideal biosensors, since they can penetrate skin and other tissues without causing harm or sensation to the patient. Due to that, several applications, in many fields of medicine, have been described [202,203]. One example can be found in the paper by Raoof et al. [204], which used several nucleic acids as probes on MWCNT electrodes, for the in vivo electrochemical determination of Hg^2+^ and Ag^+^, in dental amalgam filling compositions. Cui and coworkers [205] described a similar DNA–MWCNT electrode for the non-invasive detection of helicobacter pylori in dental plaque.

An important aspect to be considered when graphene and nanotubes-based nanomaterials are used in dental materials is biocompatibility, necessary to understand the relationships of these materials with living cells. Since nanomaterials, as exposed, are becoming very popular in medicine, several studies have been performed in order to evaluate this important aspect. In the case of nanomaterials, such as graphene and nanotubes, the biocompatibility depends on several factors such as size, purity, shape and number of sharp edges, as it is difficult to assess the general arguments [206,207]. The studies focused on the toxicity of oral applications are limited [208]. Olteanu et al. [209] performed a study to evaluate the toxicity of graphene-based materials on human dental follicle stem cells. They found 40 ug/mL as a threshold, for higher concentration, reported that the cells viability was reduced. They reported a good safety profile for low concentrations (below 5 ug/mL). In another work, Jin and coworkers [210] proposed a functionalization with polyethylene glycol or hydroxyapatite to improve the solubility and biocompability of MWCNTs for oral applications.

## 5. Conclusions, Perspectives and Future Challenges

**Figure 4:**
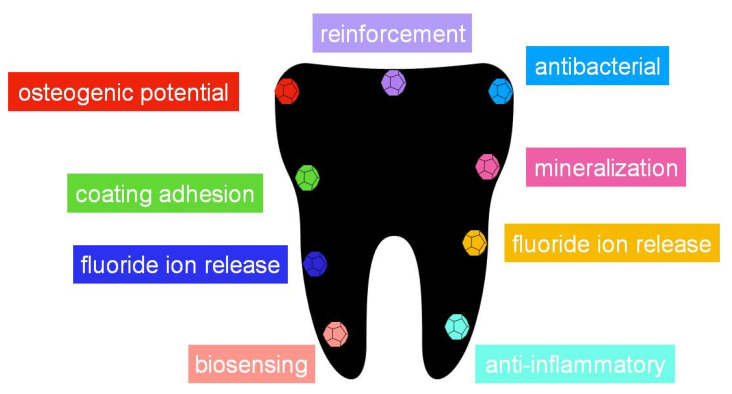
Some of the most important properties that can be enhanced in dentistry with the use of a nanomaterials.

Table 2 summarizes the main applications that nanomaterials present in the most common dentistry applications. All of them present interesting applications in some fields (Figure 4), demonstrating the potential of these materials to improve the characteristics and properties of the materials used in dentistry, such as resins, cements, implants and composites, and also their relevance for the design of new processes for therapeutic treatments and biosensing. However, among them, it seems that graphene and nanotubes, those with carbon composition, seem to be the most promising, especially graphene. As exposed, this is due to the biocompatibility of carbon materials, and to the special structure that they have. The number of papers devoted to both of them (Figure 2) indicate that, twenty years ago, most efforts were devoted to the applications of nanotubes, however, during the last years, research groups have focusing their interest in graphene applications. Since graphene was just isolated for the first time in 2004, it is nowadays when the applications in dentistry are being developed, once that other important issues, such as the synthesis and characterization methods, have been correctly described. As has been discussed, graphene can present better properties than nanotubes due to the sheet structure, especially for mechanical strength and functionalization. Thus, it is expected that this interest in graphene-based material will continue increasing in dentistry, and in the next years, more applications will be described.

Zeolites and other ordered porous materials are also very useful, as has been exposed. These materials can be used in drug delivery, and for some applications their behavior can be quite similar to graphene-based materials, with the advantage that they are less expensive, and easier to synthesize and characterize. Due to this, zeolites and MOFs have been used in many fields of medicine and it is surprising that the low amount of papers devoted to the study of their applications in dentistry (Figure 2), and subsequently, it is also expected that studies will explore them.

## Figures and Tables

**Table 1:** Main applications of zeolite-based materials in dentistry.

	Material	Main Function	References
**Ca/zeolite**	resins	remineralize	[21]
**Chlorhexidine/zeolite**	cement	antimicrobial	[24]
**Ag/zeolite**	cement	antimicrobial	[33,34,35]
	acrylic resins	antimicrobial	[45]
**Zeolite coating**	Ti alloys	osseointegration	[49]

**Table 2:** Most important applications developed in dentistry with the use of nanomaterials. GO (Graphene Oxide), NPs (NanoParticles), HA (Hydroxyapatite).

	Restorative Dentistry	Endodontics	Periodontics	Tissue Engineering	Ti Dental Implants
**Zeolites**	Incorporated to fillers when they are loaded with Ca^2+^, to confer anticaries activity. Ag/zeolite materials can be incorporated to fillers (antibiofilm capacity).	Can incorporate Ca^2+^ cations and them be incorporated to cements, for enhance their biomineralization activity. Can be loaded with drugs/Ag NPs to confer/enhance antimicrobial/autoinflammatory properties to cements.		Zeolites–HA composites have been described as active in the proliferation of osteoblast.	Coatings to increase osseointegration. Functionalized coatings with antibacterial agents.
**Graphene**	Reinforcing filler. Ag/GO additive to fillers to improve the antibiofilm capacity. GO quantum dot coatings for dentin hypersensitivity dentin treatments.	Reinforcement of cements. Ag/GO to confer/enhance antimicrobial/anti-inflammatory properties to cements.	GO/HA enhance Ca incorporation. Confer antimicrobial properties when is loading	Scaffolds. Provide suitable environment for cell incorporation and growing factors. Functionalization with proteins/peptides to enhance cell growth.	GO and GO/HA coatings enhance the osseointegration, antibacterial activity and mechanical properties; preventing corrosion.
**Carbon Nanotubes**	Reinforcing filler.	Reinforcement of cements. Incorporation of functionalized nanotubes to cement for drug delivery.		Scaffolds. Provide suitable environment for cell incorporation and growing factors.	Coatings to increase the resistance to corrosion and the osseointegration.
**Ti Nanotubes**					Coatings to increase the surface roughness in order to improve the osseointegration. Functionalized coatings with proteins/hydroxyapatite to enhance osseointegration. Functionalized coatings with anti-inflammatory or antibacterial agents.
**Hydroxyapatite/Chitosan nanorods**	Enhance the biomineralization of enamel. Fluoridated hydroxyapatite nanorods for caries Enhance the biomineralization of cements and act as reinforcement materials. prevention treatments.		Enhance Ca incorporation. Confer antimicrobial properties when is loading with Zn particles.	Scaffolds. The nano-shape enhanced their osteogenic and cell differentiation potential.	
